# Adherence to Antidiabetic Medication and Cardiovascular Outcomes in Cancer Patients: A Nationwide Population-Based Cohort Study

**DOI:** 10.3390/cancers17071117

**Published:** 2025-03-26

**Authors:** Mi-Hyang Jung, Moon-Kyung Jung, Eui-Soon Kim, Jong-Chan Youn, Eun Young Lee, Dongwoo Kang, Dae-Sung Kyoung, Woo-Baek Chung, Hae Ok Jung, Sang-Hyun Ihm, Hokyou Lee, Choon Ta Ng, Hyeon Chang Kim

**Affiliations:** 1 Division of Cardiology, Department of Internal Medicine, Seoul St. Mary’s Hospital, College of Medicine, The Catholic University of Korea, 222, Banpo-daero, Seocho-gu, Seoul 06591, Republic of Korea; floria0515@gmail.com (M.-H.J.); sky92star@gmail.com (M.-K.J.); euisoon123@gmail.com (E.-S.K.); peace816@catholic.ac.kr (W.-B.C.); hojheart@gmail.com (H.O.J.); 2 Catholic Research Institute for Intractable Cardiovascular Disease, College of Medicine, The Catholic University of Korea, Seoul 06591, Republic of Korea; limsh@catholic.ac.kr; 3 Division of Endocrinology and Metabolism, Department of Internal Medicine, Seoul St. Mary’s Hospital, College of Medicine, The Catholic University of Korea, Seoul 06591, Republic of Korea; leyme@catholic.ac.kr; 4 Data Science Team, Hanmi Pharm. Co., Ltd., Seoul 05545, Republic of Korea; dongwoo.kang94@hanmi.co.kr (D.K.); kdis97@hanmi.co.kr (D.-S.K.); 5 Division of Cardiology, Department of Internal Medicine, Bucheon St. Mary’s Hospital, College of Medicine, The Catholic University of Korea, 327, Sosa-ro, Wonmi-gu, Bucheon-si 14647, Republic of Korea; 6 Department of Preventive Medicine, Yonsei University College of Medicine, 50, Yonsei-ro, Seodaemun-gu, Seoul 03722, Republic of Korea; hokyou.lee@yuhs.ac (H.L.); hckim14@gmail.com (H.C.K.); 7 Cardiovascular and Metabolic Disease Etiology Research Center, Yonsei University College of Medicine, Seoul 03722, Republic of Korea; 8 Department of Cardiology, National Heart Centre Singapore, Singapore 169609, Singapore; choonta@gmail.com

**Keywords:** cancer, diabetes, adherence, survivorship, cardiovascular disease

## Abstract

Background/Objectives: Cancer survivors increasingly face cardiovascular disease (CVD), with diabetes as a major risk factor. This study investigates the relationship between adherence to antidiabetic medications and cardiovascular (CV) outcomes in cancer patients. Methods: Using data from the Korean National Health Insurance Service—National Sample Cohort, we assessed the clinical outcomes of adult cancer patients prescribed antidiabetic medications. The study population was categorized based on medication adherence, as determined by the medication possession ratio (MPR): good (MPR ≥ 0.8), moderate (0.5 ≤ MPR < 0.8), and poor (MPR < 0.5) adherence. The primary outcomes included overall and CV mortality, with secondary outcomes encompassing hospitalization for major CVDs and healthcare costs. Results: Among the 7928 cancer patients with diabetes, 59.7% were nonadherent (22.1% with moderate and 37.6% with poor adherence). Over a median follow-up of 5.7 years, 1462 deaths and 2897 CV events were confirmed. Multivariable adjusted analyses showed a 1.70-fold and 2.11-fold higher risk of overall mortality for the moderate and poor adherence groups, respectively, compared to the good adherence group. For CV mortality, moderate adherence was associated with a 1.51-fold higher risk, and poor adherence with a 2.10-fold higher risk. The risk for new-onset CV events increased by 1.32-fold in the moderate adherence group and 1.44-fold in the poor adherence group. The good adherence group also showed significantly lower total healthcare costs compared to the nonadherence group. Conclusions: The present study underscores the importance of adherence to antidiabetic medications in cancer patients, revealing significant associations with decreased mortality, CV events, and healthcare costs.

## Simple Summary

Cancer survivors are increasing, and diabetes is a common comorbidity among them. This study aimed to assess how well cancer patients with diabetes adhere to their antidiabetic medications and the potential impact of poor adherence on health outcomes. We found that a substantial proportion—three out of five patients—did not take their antidiabetic medications as prescribed. Poor medication adherence was linked to higher risks of death, cardiovascular disease, and increased healthcare costs. These findings highlight the urgent need for better strategies to support antidiabetic medication adherence in cancer patients to improve health outcomes and reduce healthcare burdens.

## 1. Introduction

Cancer remains a leading cause of death and a critical global health concern [1]. In recent years, cancer survival rates have notably increased due to advances in early detection and treatment. In the United States, the number of cancer survivors was 18.1 million as of January 2022, and this figure is projected to reach 22.5 million by 2032 [2]. Similarly, in the UK, cancer survival rates have doubled over the past four decades, with recent data indicating that 50% of cancer patients now live at least a decade after diagnosis [3]. As survival rates improve, greater emphasis is placed on the long-term health and well-being of cancer survivors [4,5]. However, these survivors continue to face the challenge of cardiovascular disease (CVD), the second leading cause of death in cancer patients, following only cancer itself [6]. The frequent coexistence of these diseases is driven by shared risk factors, such as smoking, alcohol consumption, and physical inactivity, as well as cancer biology, including low-grade inflammation and cardiovascular (CV) toxicity from cancer therapies [3,4,5,6,7,8,9,10]. Therefore, proactive CVD screening and proper management of its risk factors are crucial for improving the health span of cancer patients.

Diabetes, a major risk factor for CVD, is a common comorbidity in cancer patients, affecting up to one in four individuals with cancer [11,12]. Comorbid diabetes significantly increases the risk of complications such as CVD and infection, which can directly lead to death or hospitalization. Additionally, these complications may indirectly impact cancer treatment by causing disruptions or suboptimal management, ultimately worsening cancer outcomes [13,14]. Thus, comprehensive diabetes management is an essential aspect of cancer care. A key component of diabetes management is adherence to antidiabetic medication, which is crucial for preventing CV complications and improving survival. However, real-world data on antidiabetic medication adherence in cancer patients and its clinical impact remain limited [15,16,17,18]. One study reported a significant decline in antidiabetic medication adherence following a cancer diagnosis, with a 6.3% reduction at diagnosis and a continued monthly decline of −0.20% [15]. However, most prior studies focused solely on new users of antidiabetic agents, excluding prevalent users [15,16]. Additionally, these studies primarily assessed general outcomes, such as overall survival or all-cause hospitalization, and did not comprehensively evaluate CV-specific outcomes, including CV mortality or major CV events, as distinct endpoints [15,16,17]. To our knowledge, no study has directly examined the impact of nonadherence to antidiabetic medication on CV outcomes in cancer patients.

From this perspective, our study aims to investigate the association between antidiabetic medication adherence and key clinical outcomes, including all-cause mortality, CV mortality, and major CV events, in cancer patients with concurrent diabetes (both pre-existing and newly diagnosed cases). Furthermore, we seek to evaluate the economic impact of antidiabetic medication adherence by analyzing healthcare costs in this patient population.

## 2. Materials and Methods

### 2.1. Ethical Statement

The study protocol received approval from the Institutional Review Board of Severance Hospital (4-2015-0140), which waived the need for informed consent due to the anonymization of the data provided by the National Health Insurance Service (NHIS).

### 2.2. Study Design and Study Participants

We utilized the Korean NHIS—National Sample Cohort (NSC) database. The NHIS in Korea is a compulsory health insurance system covering the entire population [18]. The NHIS-NSC serves as a representative nationwide population-based cohort established to give researchers and policymakers comprehensive information about health insurance usage and services among citizens. To create this cohort, a systematic stratified random sampling technique with proportional allocation was employed. The total Korean population in 2002 was categorized into 1476 strata based on age group, sex, eligibility status, and income level. Within each stratum, individuals were sorted by total annual medical expenses, and a 2.2% sampling rate was applied to select a representative sample. This approach was used to correct for the highly skewed distribution of healthcare expenditures and to enhance the representativeness of the cohort. The selected participants were then longitudinally tracked until 2013. This database encompasses demographic characteristics, diagnostic codes, prescriptions, and inpatient and outpatient visit details, providing a valuable resource for population-based health research [18].

**Figure 1:**
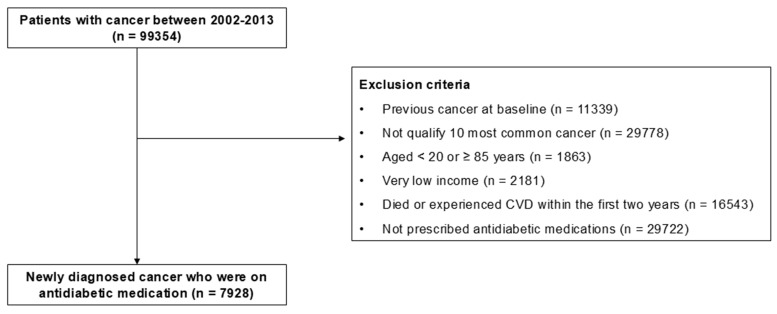
Flowchart of study population enrollment. Abbreviations: CVD, cardiovascular disease.

We collected information from the NHIS-NSC 2002–2013 database, focusing on patients diagnosed with cancer (n = 99,354). Our present analysis considered the ten most prevalent cancers in adults using ICD-10 codes (Table S1). Throughout this process, we excluded those with other forms of cancers (n = 29,778). Furthermore, to narrow down our study population to those newly diagnosed with cancer, we excluded the patients with previous cancer at the study’s outset in 2002 (n = 11,339). To minimize confounders, we excluded individuals aged < 20 years or ≥85 years (n = 1863) as well as those with a very low income (n = 2181). The very low-income group refers to medical aid recipients, who account for 3% of the total population in South Korea. Given the well-established association between low income and poor medication adherence, this extreme subgroup was excluded. To mitigate the bias of reverse causality, we also removed individuals who died or experienced CVD within the initial two years of this study (n = 16,543). Moreover, individuals who had not been prescribed antidiabetic medications throughout the entire study period were excluded (n = 29,722). Ultimately, this study comprised 7928 individuals (Figure 1).

### 2.3. Assessing Adherence to Antidiabetic Medication

We evaluated adherence to antidiabetic medication using the medication possession ratio (MPR) [16,17], calculated as the total days of supplied medication divided by the refill period. We categorized the participants into three groups based on their MPR scores—good (MPR ≥ 0.8), moderate (0.5 ≤ MPR < 0.8), and poor (MPR < 0.5)—using a classification previously employed in study [16] to facilitate the assessment of a dose–response relationship. Nonadherence was defined as an MPR below 0.8, in line with earlier studies [19,20].

### 2.4. Determining Study Outcome

Our primary study objectives centered on all-cause and CV mortality. We identified these outcomes by cross-referencing mortality records from the National Statistical Office of Korea with the NHIS database. Our secondary objectives included various CV events, such as hospitalizations related to ischemic heart disease (IHD, I20–I25), peripheral artery disease (PAD, I70–I79), a cerebrovascular accident (CVA, I60–I69), and heart failure (HF, I40–I43, I50–I52). Additionally, we used ICD-10 codes (N18, N19) to identify chronic kidney disease (CKD). To account for prior CVD history, we employed two approaches: initially, adjusting for previous CVD occurrences in the entire population (outpatient or inpatient visits due to IHD, PAD, CVA, and HF); subsequently, excluding individuals with prior CVD-related admissions and conducting the same analyses. For the latter method (the results post-exclusion of those with earlier CVD admission history), we further controlled previous outpatient visits for CVD, acknowledging that some patients might still have outpatient CVD records. This study monitored individuals from cancer diagnosis until reaching primary or secondary endpoints or study completion on 31 December 2013.

### 2.5. Statistical Analyses

Categorical variables were presented as numbers with corresponding percentages. To assess the hazards related to nonadherence to antidiabetic medication, we developed a Cox proportional hazard regression model. Hazard ratios (HRs) with 95% confidence intervals (CIs) were calculated both in crude and adjusted forms, accounting for potential confounders such as age, sex, residential area, income status, and CVD history. We ensured the proportional hazards assumption by conducting log–log plots, confirming no violation of this assumption across various outcomes concerning the MPR. Kaplan–Meier curves were utilized to illustrate the cumulative incidence of primary outcomes. Differences in primary outcomes among the groups were evaluated using the log-rank test. For the all-cause mortality analysis, subgroup evaluations were performed based on sex (male and female) and age (<60 years and ≥60 years). As an ancillary analysis, we examined healthcare expenditures in relation to adherence to antidiabetic medication. Initially, the total healthcare cost was computed as the sum of all healthcare insurance reimbursement costs from the initial diagnosis of diabetes for each individual until the end of the study period. Subsequently, healthcare cost per individual was determined by dividing the total healthcare cost by the number of individuals. An ANOVA test was utilized to compare the differences in healthcare expenditure among the three groups. A post hoc analysis was performed using the Bonferroni test. We set the threshold for statistical significance at a *p*-value of <0.05. All statistical analyses were conducted using SAS version 9.4 (SAS Institute Inc., Cary, NC, USA).

## 3. Results

### 3.1. Baseline Characteristics

In this study, a total of 7928 cancer patients with diabetes were included, and their baseline characteristics are outlined in Table 1. Among the participants, 32.1% were female, 59.1% were aged 60 or above, and 57.6% had pre-existing diabetes. In terms of adherence to antidiabetic medications, 40.3% belonged to the good adherence group, while 59.7% were categorized as a non-adherence group (with 22.1% demonstrating moderate adherence and 37.6% having poor adherence). The prevalence of non-adherence was higher in those newly diagnosed with diabetes than in those with pre-existing diabetes (65.4% and 55.5%, respectively). Throughout a median follow-up period of 5.7 years, there were 1462 deaths, and 2897 reported cardiovascular events.

### 3.2. Primary Outcomes: Association of Antidiabetic Medication Adherence and All-Cause and CV Mortality

In comparison to the good adherence group, both the moderate and poor adherence groups exhibited a 1.70-fold and 2.11-fold increase in the risk of all-cause mortality, respectively, after adjusting for potential confounding factors (adjusted HR 1.70 [95% CI, 1.47–1.98] for the moderate adherence group, and adjusted HR 2.11 [95% CI, 1.86–2.40] for the poor adherence group; Table 2). Specifically, regarding CV mortality, the moderate and poor adherence groups demonstrated a 1.51-fold and 2.10-fold heightened risk, respectively, when compared to the good adherence group (adjusted HR 1.51 [95% CI, 1.01–2.27] for the moderate adherence group and adjusted HR 2.10 [95% CI, 1.50–2.95] for the poor adherence group).

**Figure 2:**
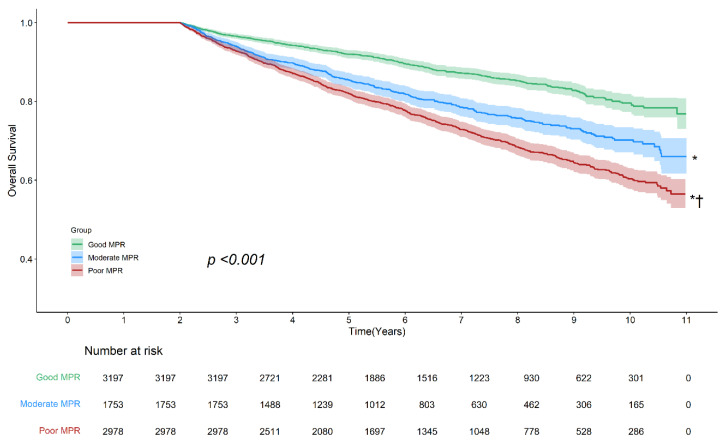
Kaplan–Meier survival curves illustrating overall survival based on adherence to antidiabetic medications. The Kaplan–Meier curve demonstrated a significant difference in survival rates across adherence groups (log-rank *p* < 0.001), with the highest survival rate observed in the good adherence group, followed by the moderate and poor adherence groups. Pairwise comparisons showed statistically significant survival differences between all three groups. Asterisk (*) indicates *p* < 0.001 vs. good adherence; dagger (†) indicates *p* < 0.001 vs. moderate adherence. Shaded areas represent 95% confidence intervals. Abbreviations: MPR, medication possession ratio.

The Kaplan–Meier curve revealed a risk gradient based on the level of antidiabetic medication adherence (log-rank *p* < 0.001). The good adherence group showed the highest survival rate, followed by the moderate and poor adherence groups. Although the moderate and poor adherence groups initially showed overlapping survival rates, distinct risk differences emerged over time (Figure 2). The subgroup analyses by age and sex were generally similar to the main analyses, except for the weakened risk discrimination between the moderate and poor adherence groups (Figures S1 and S2, Table S2).

### 3.3. Secondary Outcomes: Association of Antidiabetic Drug Adherence with a CVD Occurrence

Compared to the good adherence group, both the moderate and poor adherence groups exhibited a 1.32-fold and 1.44-fold increased risk for any CVD, respectively, after controlling for covariates (adjusted HR 1.32 [95% CI, 1.19–1.45] for the moderate adherence group, and adjusted HR 1.44 [95% CI, 1.32–1.56] for the poor adherence group) (Table 3). Consistent findings supporting the risk associated with non-adherence to antidiabetic medications were observed for each specific subtype of CVD, such as IHD, PAD, CVA, and HF (Table 3).

Furthermore, when repeating the same analyses after excluding those with a prior history of a myocardial infarction, a stroke, heart failure, and chronic kidney disease (conducted as a sensitivity analysis), similar findings were obtained (Table S3).

### 3.4. Ancillary Analysis: Healthcare Expenditure Based on Antidiabetic Medication Adherence

**Figure 3:**
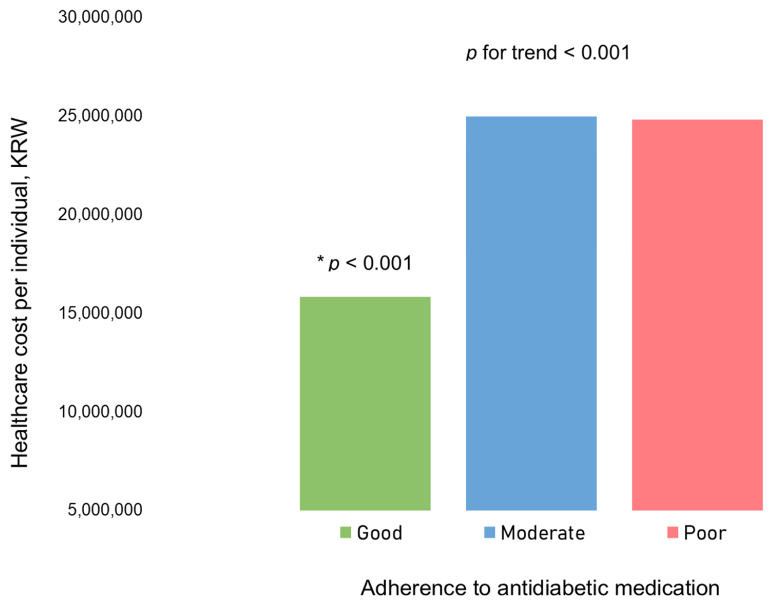
Healthcare cost per individual based on adherence to antidiabetic medications. The bars represent the mean healthcare cost per individual for each adherence category. There was a significant difference in healthcare cost per individual based on adherence to antidiabetic medications (*p* for trend < 0.001). Specifically, the group with good adherence showed a significantly lower healthcare cost per individual than the two other groups (moderate and poor adherence groups), as indicated by post hoc analysis (presented as an asterisk). As of 30 January 2024, USD 1 = KRW 1330.

In our examination of healthcare costs per individual based on antidiabetic medication adherence, the good adherence group demonstrated significantly lower medical expenses compared to the other groups (Figure 3, Table S4).

## 4. Discussion

In this population-based cohort study involving individuals with concurrent cancer and diabetes, we observed a potential association between adherence to antidiabetic medication and clinical outcomes. Notably, the prevalence of nonadherence to antidiabetic medications was substantial among cancer patients, affecting three in five individuals. We observed a gradual increase in the risk of all-cause and CV mortality associated with varying levels of adherence to antidiabetic medication. Regarding the occurrence of new-onset CVD, non-adherence to antidiabetic drugs was associated with the development of both individual and composite CVD. Furthermore, nonadherence to antidiabetic medication was linked to increased healthcare costs. Collectively, our study underscores the importance of clinical attention to ensure better adherence to antidiabetic medications among cancer patients with diabetes, aiming to prevent adverse outcomes.

Numerous studies highlight the significant role of adherence to antidiabetic medication in glycemic control and reducing micro- and macro-vascular complications [21,22,23,24,25,26]. A recent meta-analysis involving eight observational studies reported that good adherence to antidiabetic medication is associated with a reduced risk of all-cause mortality and hospitalization in individuals with type 2 diabetes [27]. Good adherence was linked to a 28% decreased risk for all-cause mortality and a 10% decreased risk for hospitalization [27]. Furthermore, several studies have consistently shown that non-adherence to antidiabetic medications is associated with increased healthcare costs, corroborating our study’s findings [28,29]. The higher healthcare costs in the non-adherent group may be attributed to elevated hospitalization rates related to various diabetic complications. However, the previous studies were mainly conducted on the general population, without the specific inclusion or specification of individuals with cancer. The significance of our current study lies in expanding the previous research, emphasizing that the importance of adherence to antidiabetic medication also applies to patients with cancer, who are considered a vulnerable population.

Adherence to antidiabetic medication is generally low in the general population [27,28], and this pattern is also observed in cancer patients, with some studies reporting similarly low adherence rates [16] and others suggesting even lower adherence compared to the general population [30,31,32]. In a meta-analysis of diabetes medication adherence in the general population, the prevalence of nonadherence (MPR < 0.8) was 37.8% [27], which is lower than the 59.7% observed in our study, highlighting the greater challenges in medication adherence among cancer patients. A prior study found that diabetes medication adherence among breast cancer survivors was lower than that observed for other chronic conditions, such as hypertension or dyslipidemia [31]. Additionally, another study demonstrated that diabetes in cancer survivors was managed less proactively compared to diabetes in individuals with other chronic diseases [32]. A previous study using the MarketScan Claims database examined adult cancer patients (breast, prostate, colon, lung) who were new users of antidiabetic medications and found suboptimal adherence, with only 38% classified as adherent. However, the adherence levels were similarly low in the matched control group [16], possibly due to the study’s restriction to new users of antidiabetic medications. Consistent with our findings, nonadherence was associated with higher hospitalization rates. However, unlike our study, they did not observe a significant impact on total medical costs [16]. These differences may be attributed to variations in study populations: our study included a broader range of cancers and both prevalent and newly diagnosed diabetes in adults aged 20–84 years, whereas their study focused only on new users, included patients aged 18–64 years, and examined four representative cancers. Interestingly, their findings also indicated that a younger age was associated with poorer medication adherence, which aligns with our results. Moreover, they observed that breast and prostate cancer patients exhibited better adherence compared to those with colon and lung cancer. Another study on colorectal cancer patients with diabetes demonstrated that good adherence to antidiabetic medication was associated with a reduced risk of death; yet, the prevalence of good adherence was only 22.5% [17]. Similarly, Calip et al. reported a decline in antidiabetic medication adherence after a breast cancer diagnosis [31]. A study from the Netherlands also found a decrease in adherence following a cancer diagnosis [15]. These declines in adherence may stem from various factors, including the prioritization of cancer treatment, reduced oral intake, metabolic changes, or physician recommendations to discontinue antidiabetic medication. However, these declines may also contribute to worsened metabolic control, increased CV risk, and poorer overall survival. Furthermore, a previous cohort study in cancer survivors found that the optimal glucose range for survival was relatively narrow (85–99 mg/dL), similar to that observed in the general population. This finding highlights the importance of regular monitoring and meticulous glucose management, even in cancer patients [33].

Our findings strongly support the notion that maintaining quality control in diabetes, particularly through ensuring good adherence to antidiabetic medication, is crucial for preventing adverse clinical outcomes including overall and CV mortality, as well as hospitalization due to CVD. Additionally, it contributes to the reduction of healthcare costs among patients with cancer. Improving medication adherence can be achieved firstly by recognizing the risk of nonadherence and secondly by attentively assessing and confirming the status of drug adherence in each patient. In addition, simplifying medication regimens, educating patients and their caregivers, using digital reminders, and ensuring regular follow-ups can further enhance adherence [34,35].

This study has several inherent limitations. First, due to its retrospective nature, establishing causal inferences between medication adherence and clinical outcomes remains challenging due to potential confounding factors. Our data lacked important CV risk variables, such as smoking history and alcohol consumption, which may have influenced both medication adherence and CVD outcomes. Second, medication adherence was assessed using prescription claims data rather than direct patient-reported measures or electronic monitoring devices. As claims data do not confirm whether medications were actually taken as prescribed, this may lead to misclassification. Third, our dataset did not include specific numerical values for blood glucose control, such as hemoglobin A1c, limiting our ability to assess glycemic control in relation to CVD risks. Fourth, we did not differentiate between antidiabetic medication classes due to the small sample sizes within each drug class, despite their potentially distinct CV effects. Additionally, we did not assess whether patients were on a single medication or multiple medications. Given that regimen complexity can negatively impact adherence, future studies should examine the effects of specific drug classes and polypharmacy on adherence and clinical outcomes. Fifth, this study focused on patients with the ten most common cancers rather than including all cancer types. Our findings may not be fully generalizable to less prevalent cancers with different treatment regimens or metabolic effects. Future research should incorporate broader cancer cohorts with more detailed information on cancer stage, histologic subtype, and treatment modalities to provide a more comprehensive analysis. Sixth, this study was conducted in a single country, South Korea, and thus the generalizability of our findings to other populations, including different ethnic groups and geographic regions, remains uncertain. Differences in healthcare systems, socioeconomic factors, and genetic predispositions may influence medication adherence patterns and CVD outcomes in cancer patients with diabetes. Seventh, our study did not focus exclusively on cancer survivors in remission (typically defined as disease-free survival beyond five years) [4,35]. Instead, our cohort included patients at various stages of their cancer journey, including those newly diagnosed, undergoing treatment, and in remission. Since cancer survivors with stable disease represent a distinct subgroup, future research specifically targeting this population may provide further insights into the long-term impact of medication adherence on CVD risks in this important subgroup. Finally, while we employed the Cox proportional hazard model for its interpretability and clinical applicability, future studies may benefit from incorporating a deep learning-based survival analysis or machine learning models. These approaches could help uncover complex, nonlinear interactions in medication adherence patterns and CVD outcomes. Despite these limitations, our findings provide valuable real-world evidence in an understudied and often overlooked patient population. Given that conducting a randomized controlled trial to assess the long-term impact of antidiabetic medication adherence in cancer patients with diabetes is logistically and ethically impractical, our study offers meaningful clinical insights into the long-term consequences of adherence in this vulnerable patient group.

## 5. Conclusions

The present study highlights the concerning clinical issue of nonadherence to antidiabetic medications among cancer patients, with a substantial proportion—three in five—found to not adhere to their prescribed antidiabetic drugs. This poor adherence status underscores the critical importance of adherence to these medications, as significant associations were identified with increased mortality, CV events, and healthcare costs. Addressing this alarming trend of nonadherence among cancer patients is crucial for improving outcomes. Focusing on enhancing adherence to antidiabetic medications in this population is imperative to mitigate these risks and improve overall health outcomes.

## Figures and Tables

**Table 1:** Baseline characteristics.

Variable		Total, n	Good Adherence	Moderate Adherence	Poor Adherence
Participants	Number	7928	3197 (40.3%)	1753 (22.1%)	2978 (37.6%)
Sex	Male	5382	2112 (39.2%)	1202 (22.3%)	2068 (38.4%)
	Female	2546	1085 (42.6%)	551 (21.6%)	910 (35.7%)
Age group, years	20–39	263	66 (25.1%)	50 (19.0%)	147 (55.9%)
	40–59	2977	1182 (39.7%)	677 (22.7%)	1118 (37.6%)
	60–84	4688	1949 (41.6%)	1026 (21.9%)	1713 (36.5%)
Residential area	Seoul	1679	712 (42.4%)	338 (20.1%)	629 (37.5%)
	Urban	1963	780 (39.7%)	433 (22.1%)	750 (38.2%)
	Suburb	4286	1705 (39.8%)	982 (22.9%)	1599 (37.3%)
Income level	≤30% (low)	1516	583 (38.5%)	347 (22.9%)	586 (38.7%)
	31–60%	1911	750 (39.2%)	436 (22.8%)	725 (37.9%)
	61–90%	2975	1207 (40.6%)	639 (21.5%)	1129 (37.9%)
	91–100% (high)	1526	657 (43.1%)	331 (21.7%)	538 (35.3%)
Prevalence of cancer	Breast cancer	279	153 (54.8%)	54 (19.4%)	72 (25.8%)
	Non-Hodgkin’s disease	58	24 (41.4%)	16 (27.6%)	18 (31.0%)
	Gastric cancer	1137	417 (36.7%)	245 (21.5%)	475 (41.8%)
	Colon cancer	1322	559 (42.3%)	290 (21.9%)	473 (35.8%)
	Lung cancer	1049	417 (39.8%)	227 (21.6%)	403 (38.4%)
	Renal cell carcinoma	152	63 (41.4%)	36 (23.7%)	53 (34.8%)
	Liver cancer	2018	705 (34.9%)	470 (23.3%)	843 (41.8%)
	Gallbladder cancer	104	50 (48.1%)	21 (20.2%)	33 (31.7%)
	Ovarian cancer	179	68 (38.0%)	43 (24.0%)	68 (38.0%)
	Prostate cancer	1630	739 (45.3%)	351 (21.5%)	540 (33.1%)
Previous medical history ^a^	Any CVD	4047	1678 (41.7%)	959 (23.7%)	1410 (34.8%)
	IHD	2358	1010 (42.8%)	567 (24.0%)	781 (33.1%)
	PAD	2174	926 (42.6%)	504 (23.2%)	744 (34.2%)
	CVA	1473	588 (39.9%)	356 (24.2%)	529 (35.9%)
	HF	834	297 (35.6%)	198 (23.7%)	339 (40.6%)
	CKD	271	73 (26.9%)	58 (21.4%)	140 (51.7%)
Previous admission history ^b^	Any CVD	1210	465 (38.4%)	272 (22.5%)	473 (39.1%)
	IHD	762	294 (38.6%)	173 (22.7%)	295 (38.7%)
	PAD	208	76 (36.5%)	43 (20.7%)	89 (42.8%)
	CVA	484	164 (33.9%)	117 (24.2%)	203 (41.9%)
	HF	215	69 (32.1%)	48 (22.3%)	98 (45.6%)
	CKD	122	18 (14.8%)	25 (20.5%)	79 (64.8%)
Anti-diabetic medication	AGI	1623	866 (53.4%)	460 (28.3%)	297 (18.3%)
	SU	3884	1850 (47.6%)	1094 (28.2%)	940 (24.2%)
	GLP-1	-	- (0.0%)	- (0.0%)	- (0.0%)
	DPP4I	235	144 (44.3%)	66 (28.1%)	25 (10.6%)
	INSULIN	1505	539 (35.8%)	413 (27.4%)	553 (35.4%)
	MET	3280	1653 (49.8%)	894 (27.3%)	733 (22.3%)
	GLN	428	210 (49.1%)	141 (32.9%)	77 (18.0%)
	TZD	821	453 (53.0%)	235 (28.6%)	133 (16.2%)
Status of diabetes	Pre-existing	4564	2032 (44.5%)	1213 (26.6%)	1319 (28.9%)
	Newly diagnosed	3364	1165 (34.6%)	540 (16.1%)	1659 (49.3%)

Percentages in each adherence category (good, moderate, and poor) represent the proportion of individuals within each subgroup classified by adherence level. Abbreviations: CVD, cardiovascular disease; IHD, ischemic heart disease; PAD, peripheral artery disease; CVA, cerebrovascular disease; HF, heart failure; CKD, chronic kidney disease; AGI, alpha-glucosidase inhibitor; SU, sulfonylurea; GLP-1, glucagon like peptide-1 agonist; DPP4I, dipeptidyl peptidase-4 inhibitor; MET, metformin; GLN, glinides; TZD, thiazolidinedione. ^a^ Previous medical history indicates either outpatient visit or hospitalization due to corresponding diagnosis. ^b^ Previous admission history indicates hospitalization due to corresponding diagnosis.

**Table 2:** Risk of mortality related to antidiabetic medication nonadherence in cancer patients (n = 7928).

Outcome	Group	No. of Events	Crude		Multivariable Adjusted *	
			HR (95% CI)	*p* Value	HR (95% CI)	*p* Value
All-cause mortality	Good	369	1.00 (reference)	-	1.00 (reference)	-
	Moderate	339	1.72 (1.49–2.00)	<0.001	1.70 (1.47–1.98)	<0.001
	Poor	754	2.28 (2.01–2.58)	<0.001	2.11 (1.86–2.40)	<0.001
CV mortality	Good	51	1.00 (reference)	-	1.00 (reference)	-
	Moderate	43	1.58 (1.06–2.38)	0.027	1.51 (1.01–2.27)	0.046
	Poor	108	2.37 (1.70–3.31)	<0.001	2.10 (1.50–2.95)	<0.001

* Adjusted for sex, age, residential area, income level, and previous history of cardiovascular disease (either outpatient visit or hospitalization due to ischemic heart disease, peripheral artery disease, cerebrovascular accident, and heart failure), and previous history of chronic kidney disease (either outpatient visit or hospitalization due to chronic kidney disease). Abbreviations: CI, confidence interval; CV, cardiovascular; HR, hazard ratio.

**Table 3:** Risk of development of future CVD in cancer patients due to antidiabetic medication nonadherence (n = 7928).

Outcome	Group	No. of Events	Crude		Multivariable Adjusted *	
			HR (95% CI)	*p* Value	HR (95% CI)	*p* Value
New-onset any CVD	Good	998	1.00 (reference)	-	1.00 (reference)	-
	Moderate	678	1.32 (1.20–1.45)	<0.001	1.32 (1.19–1.45)	<0.001
	Poor	1221	1.43 (1.31–1.55)	<0.001	1.44 (1.32–1.56)	<0.001
New-onset IHD	Good	662	1.00 (reference)	-	1.00 (reference)	-
	Moderate	448	1.29 (1.14–1.45)	<0.001	1.28 (1.14–1.44)	<0.001
	Poor	802	1.37 (1.24–1.52)	<0.001	1.40 (1.26–1.55)	<0.001
New-onset PAD	Good	187	1.00 (reference)	-	1.00 (reference)	-
	Moderate	145	1.47 (1.18–1.82)	0.0005	1.41 (1.14–1.75)	0.0019
	Poor	245	1.47 (1.21–1.78)	<0.001	1.43 (1.18–1.73)	0.0003
New-onset CVA	Good	405	1.00 (reference)	-	1.00 (reference)	-
	Moderate	288	1.34 (1.16–1.56)	0.0001	1.32 (1.13–1.53)	0.0004
	Poor	544	1.55 (1.36–1.76)	<0.001	1.51 (1.33–1.72)	<0.001
New-onset HF	Good	197	1.00 (reference)	-	1.00 (reference)	-
	Moderate	156	1.49 (1.21–1.84)	0.0002	1.44 (1.17–1.78)	0.0007
	Poor	345	1.98 (1.67–2.36)	<0.001	1.92 (1.60–2.29)	<0.001

* Adjusted for sex, age, residential area, income level, and previous history of cardiovascular disease (either outpatient visit or hospitalization due to ischemic heart disease, peripheral artery disease, a cerebrovascular accident, and heart failure), and previous history of chronic kidney disease (either outpatient visit or hospitalization due to chronic kidney disease). Abbreviations: CI, confidence interval; HR, hazard ratio; CVD, cardiovascular disease; IHD, ischemic heart disease; PAD, peripheral artery disease; CVA, cardiovascular accident; HF, heart failure.

## Data Availability

Data are available from the NHIS for researchers with approved study proposals.

## Supplementary Materials

The following supporting information can be downloaded at https://www.mdpi.com/article/10.3390/cancers17071117/s1. Table S1. International Classification of Disease (10th edition) Clinical Modification (ICD 10-CM) codes used to define study population. Table S2. Risk of antidiabetic medication nonadherence on overall mortality in cancer patients: Subgroup analyses by age and sex. Table S3. Risk of antidiabetic medication nonadherence on future CVD occurrence in cancer patients after exclusion of those with prior admission history of IHD, PAD, CVA and CKD (n = 7039). Table S4. Healthcare expenditures based on antidiabetic medication adherence. Figure S1. Kaplan-Meier survival curves illustrating overall survival, stratified by age in relation to antidiabetic medication adherence. Figure S2. Kaplan-Meier survival curves illustrating overall survival, stratified by sex in relation to antidiabetic medication adherence.
