# Supplementary material for: Adherence to Antidiabetic Medication and Cardiovascular Outcomes in Cancer Patients: A Nationwide Population-Based Cohort Study
Source: Cancers (Basel). 2025 Mar 26;17(7):1117. doi: 10.3390/cancers17071117 (PMC11987829; doi:10.3390/cancers17071117)
Supplement: Supplementary file 1 [file cancers-17-01117-s001.zip › cancers-3526075-supplementary.pdf]

Table S1. International Classification of Disease (10th edition) Clinical Modification (ICD 10-CM) codes used to define study population

Cancer	ICD-10 codes
Breast cancer	C50
Colon cancer	C18-C20
Gastric cancer	C16
Gallbladder cancer	C23
Liver cancer	C22
Lung cancer	C34
Non-Hodgkin’s disease	C82-C86
Ovarian cancer	C56
Prostate cancer	C61
Renal cell carcinoma	C64

Table S2. Risk of antidiabetic medication nonadherence on overall mortality in cancer patients: Subgroup analyses by age and sex

Outcome	Subgroups		Group	No. of events	Crude		Multivariable-adjusted*	
					HR (95% CI)	*P* value	HR (95% CI)	*P* value
Overall mortality	Age	<60	Good	72	1.00 (reference)	-	1.00 (reference)	-
			Moderate	93	2.28 (1.68-3.10)	<0.001	2.31 (1.70-3.14)	<0.001
			Poor	223	3.26 (2.50-4.25)	<0.001	3.33 (2.55-4.35)	<0.001
		≥60	Good	297	1.00 (reference)	-	1.00 (reference)	-
			Moderate	246	1.63 (1.37-1.93)	<0.001	1.56 (1.32-1.85)	<0.001
			Poor	531	2.08 (1.81-2.40)	<0.001	1.83 (1.58-2.11)	<0.001
	Sex	Male	Good	269	1.00 (reference)	-	1.00 (reference)	-
			Moderate	231	1.53 (1.28-1.82)	<0.001	1.55 (1.30-1.85)	<0.001
			Poor	548	2.18 (1.89-2.53)	<0.001	2.09 (1.81-2.42)	<0.001
		Female	Good	100	1.00 (reference)	-	1.00 (reference)	-
			Moderate	108	2.26 (1.72-2.96)	<0.001	2.12 (1.62-2.79)	<0.001
			Poor	206	2.48 (1.95-3.14)	<0.001	2.17 (1.70-2.77)	<0.001

*Adjusted for sex, age, residential area, income level and previous history of cardiovascular disease (either outpatient visit or hospitalization due to ischemic heart disease, peripheral artery disease, cerebrovascular accident, and heart failure), and previous history of chronic kidney disease (either outpatient visit or hospitalization due to chronic kidney disease).

CI, confidence interval; HR, hazard ratio.

Table S3. Risk of antidiabetic medication nonadherence on future CVD occurrence in cancer patients after exclusion of those with prior admission history of IHD, PAD, CVA and CKD (n = 7,039).

Outcome	Group	No. of events	Crude		Multivariable-adjusted*	
			HR (95% CI)	*P* value	HR (95% CI)	*P* value
New-onset any CVD	Good	818	1.00 (reference)	-	1.00 (reference)	-
	Moderate	552	1.32 (1.19-1.47)	<0.001	1.32 (1.18-1.47)	<0.001
	Poor	940	1.39 (1.26-1.52)	<0.001	1.42 (1.29- 1.56)	<0.001
New-onset IHD	Good	543	1.00 (reference)	-	1.00 (reference)	-
	Moderate	362	1.28 (1.12-1.46)	0.0003	1.28 (1.12-1.46)	0.0003
	Poor	609	1.32 (1.18-1.48)	<0.001	1.37 (1.22-1.54)	<0.001
New-onset PAD	Good	144	1.00 (reference)	-	1.00 (reference)	-
	Moderate	121	1.61 (1.27-2.05)	0.0001	1.57 (1.23-2.00)	0.0003
	Poor	185	1.51 (1.21-1.87)	0.0002	1.48 (1.19- 1.85)	0.0005
New-onset CVA	Good	291	1.00 (reference)	-	1.00 (reference)	-
	Moderate	220	1.45 (1.22-1.73)	<0.001	1.45 (1.21-1.72)	<0.001
	Poor	384	1.56 (1.34-1.82)	<0.001	1.56 (1.33-1.82)	<0.001
New-onset HF	Good	160	1.00 (reference)	-	1.00 (reference)	-
	Moderate	110	1.30 (1.02-1.66)	0.033	1.27 (0.99- 1.61)	0.059
	Poor	247	1.82 (1.49- 2.22)	<0.001	1.78 (1.46 - 2.18)	<0.001

*Adjusted for sex, age, residential area, income level and previous history of cardiovascular disease (either outpatient visit or hospitalization due to ischemic heart disease, peripheral artery disease, cerebrovascular accident, and heart failure), and previous history of chronic kidney disease (either outpatient visit or hospitalization due to chronic kidney disease).

CI, confidence interval; HR, hazard ratio; CVD, cardiovascular disease; IHD, ischemic heart disease; PAD, peripheral artery disease; CVA, cardiovascular accident; HF, heart failure.

Table S4. Healthcare expenditures based on antidiabetic medication adherence

Variable	Total	Good	Moderate	Poor
**Number of individuals**	7,928	3,197 (40.3%)	1,753 (22.1%)	2,978 (37.6%)
**Total healthcare cost, KRW[a]**	168,321,080,660	50,582,153,120 (30.1%)	43,800,807,010 (26.0%)	73,938,120,530 (43.9%)
**Healthcare cost per individual, KRW[b]**	21,239,253	15,836,617	24,986,199	24,828,113

[a]It was estimated from the total healthcare insurance reimbursement cost.

[b]It was estimated by dividing the total healthcare insurance reimbursement cost by the number of individuals. The calculation was made after excluding those with missing medical expense data (3 people in those with good adherence). 1 USD = 1,330 KRW as of January 30th, 2024.

Figure S1. Kaplan-Meier survival curves illustrating overall survival, stratified by age in relation to antidiabetic medication adherence.

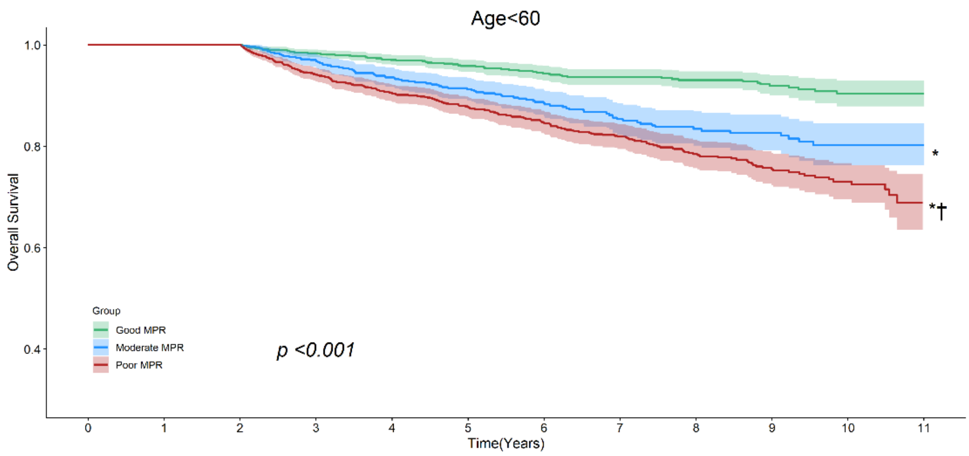

Number at risk

Good MPR	1248	1248	1248	1123	963	814	663	553	432	304	155	0
Moderate MPR	727	727	727	650	556	465	376	297	221	155	83	0
Poor MPR	1265	1265	1265	1089	929	763	627	502	381	266	149	0

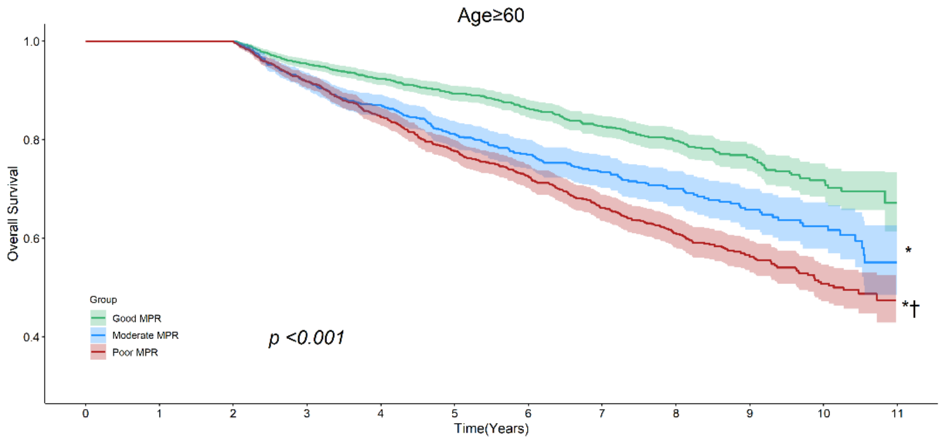

Number at risk

Good MPR	1949	1949	1949	1598	1318	1072	853	670	498	318	146	0
Moderate MPR	1026	1026	1026	838	683	547	427	333	241	151	82	0
Poor MPR	1713	1713	1713	1422	1151	934	718	546	397	262	137	0

Subgroup analyses by age similarly showed a risk gradient linked to antidiabetic medication adherence (log-rank p < 0.001), with the highest survival rate seen in the good adherence group, followed by moderate and poor adherence groups, akin to the main analyses. Asterisk (*) indicates p < 0.001 vs. good adherence; dagger (†) indicates p < 0.001 vs. moderate adherence. Shaded area represents 95% confidence intervals.

Figure S2. Kaplan-Meier survival curves illustrating overall survival, stratified by sex in relation to antidiabetic medication adherence.

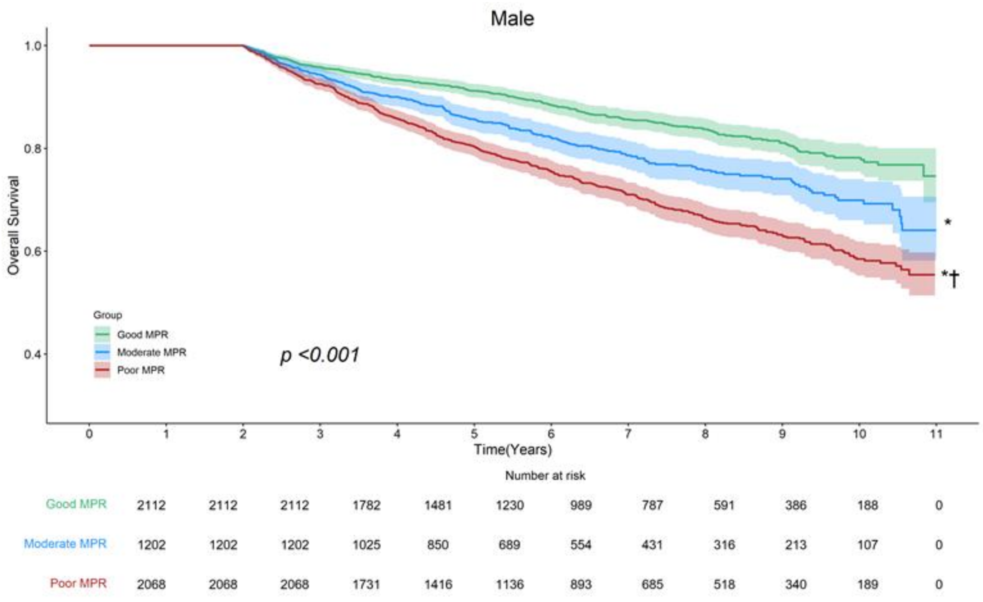

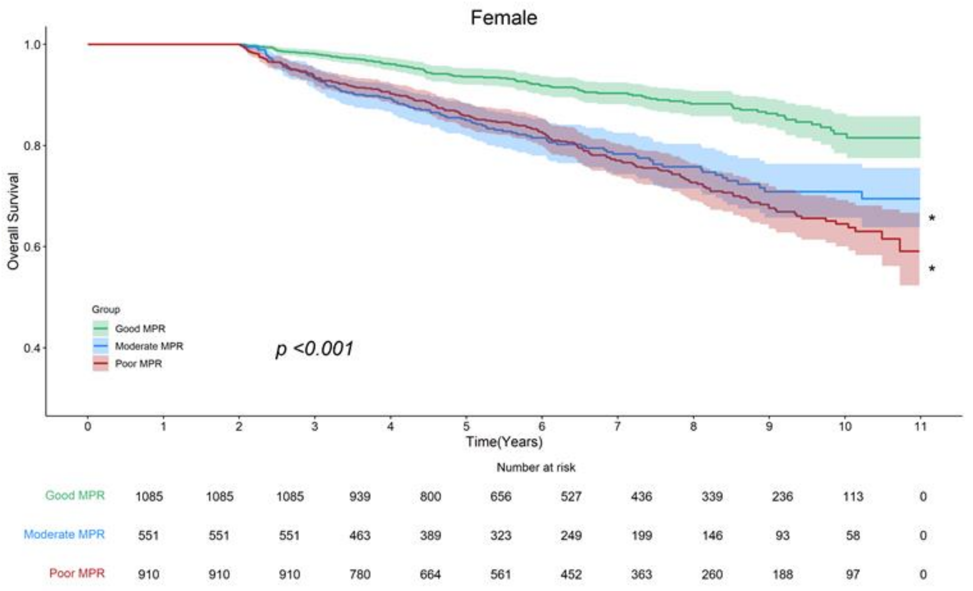

Subgroup analyses by sex exhibited a risk gradient linked to antidiabetic medication adherence (log-rank $p < 0.001$), resembling the main analyses, but with less differentiation between the moderate and poor adherence groups. Asterisk (*) indicates $p < 0.001$ vs. good adherence; dagger (†) indicates $p < 0.001$ vs. moderate adherence. Shaded area represents 95% confidence intervals.
